# LncRNA SNHG14 activates autophagy via regulating miR-493-5p/Mef2c axis to alleviate osteoporosis progression

**DOI:** 10.1038/s42003-023-05493-8

**Published:** 2023-11-04

**Authors:** Jingbo Xue, Lulu Liu, Hao Liu, Zepeng Li

**Affiliations:** The First Affiliated Hospital, Department of Spine Surgery, Hengyang Medical School, University of South China, Hengyang, 421001 Hunan Province PR China

**Keywords:** Cell division, Diseases

## Abstract

Osteoporosis is a progressive bone disease caused by impaired function of endogenous bone marrow-derived mesenchymal stem cells (BMSCs). Herein, we investigated the mechanism of lncRNA SNHG14 in osteoporosis progression. BMSCs were isolated from BALB/c mice. The osteogenic ability of BMSCs was assessed by Alkaline phosphatase (ALP) and Alizarin Red S Staining (ARS) staining. The interaction between miR-493-5p and SNHG14 or myocyte enhancer factor 2 C (Mef2c) was confirmed by dual-luciferase reporter assay. Bone histomorphometry changes were evaluated to analyze SNHG14’roles in osteoporosis in vivo. Our results illustrated *SNHG14* and *Mef2c* levels were increased in a time-dependent manner in BMSCs, and *miR-493-5p* expression was decreased. *SNHG14* knockdown inhibited osteogenic differentiation of BMSCs, and *SNHG14* upregulation had the opposite effect. *SNHG14* overexpression elevated bone mineral density and bone trabecular number, and alleviated osteoporosis progression in vivo. Mechanically, *miR-493-5p* was a target of *SNHG14*, and *miR-493-5p* targeted the *Mef2c* gene directly. *SNHG14* overexpression reversed the inhibition of *miR-493-5p* on the osteogenic ability of BMSCs, and *miR-493-5p* silencing accelerated BMSCs osteogenesis by activating Mef2c-mediated autophagy to accelerate BMSCs osteogenesis. In short, *SNHG14* activated autophagy via regulating *miR-493-5p*/*Mef2c* axis to alleviate osteoporosis progression, which might provide a new molecular target for osteoporosis treatment.

## Abstract (web summary)

SNHG14 regulates the expression of Mef2c and activates autophagy through miR-493-5p, promoting osteogenic differentiation of BMSCs and consequently alleviating osteoporosis development.

## Introduction

Osteoporosis is a systemic skeletal disease characterized by low bone density and microarchitectural deterioration of bone tissue, leading to enhanced bone fracture risk. With the increase of the aging population, osteoporosis is threatening human health, especially postmenopausal women and the elderly^1^. Osteoporosis is a growing medical, social, and economic problem because of its prevalence (affecting more than 10% of the population) and its consequences (fractures)^2^. At present, bone resorption inhibitors or bone formation enhancers are mainly used for treatment, such as bisphosphonate and intermittent parathyroid hormone. However, long-term use of drugs has side effects, and the therapeutic effect is not satisfactory^3^. Bone marrow mesenchymal stem cells (BMSCs) are multifunctional cells that can be differentiated into osteoblasts, bone cells, and adipocytes^4^. It is reported that the differentiation capacity of BMSCs is related to osteoporosis. The specific manifestation is that the decrease of osteogenic differentiation of BMSCs will cause the disorder of bone formation and promote the occurrence and development of osteoporosis^5^. Therefore, promoting osteogenic differentiation of BMSCs in a certain way will help to alleviate osteoporosis.

Long noncoding RNAs (lncRNAs) participate in several biological processes, including control of gene transcription, pre-mRNA processing, regulation of messenger RNA (mRNA) stability, and protein translation^6,7^. Meanwhile, lncRNA is involved in the regulation of an increasing number of diseases, such as osteosarcoma and Rheumatoid Arthritis, etc.^8–10^. The latest studies have shown that there are abnormal lncRNA in sequence, spatial structure, expression, and protein interaction, which have a vital effect on the pathogenesis of osteoporosis^11^. Moreover, lncRNAs act as competing endogenous RNAs (ceRNAs) to target and degrade microRNAs (miRNAs)^12^. Small nucleolar RNA host gene 14 (SNHG14), also known as UBE3A-ATS, is located on chromosome 15q11.2^13^. Du et al. showed that SNHG14 induced osteogenic differentiation of hMSC in vitro by targeting miR-2861^14^. Preliminarily, by applying miRDB software (http://www.mirdb.org/index.html), we found that there was a binding site between *SNHG14* and *miR-493-5p*. However, it was not clear whether *SNHG14* participated in the progression of osteoporosis through targeting *miR-493-5p*.

MiRNA is a noncoding RNA about 22 bp length that binds to the target mRNA 3′ - untranslated region (3′-UTR) to inhibit its levels. Increasing evidence shows that miRNA is released into bone microenvironment and participates in bone remodeling in the process of osteoporosis^15^. Further analyzing the specific mechanism, it is possible that miRNA may increase bone mineral density by accelerating the osteogenic differentiation of endogenous BMSCs^16^. Tilde et al. findings showed that overexpression of miR-138 inhibited the differentiation of hBMSCs into osteoblasts^6^. However, Yuan et al. indicated that *miR-92b-5p* could improve osteoporosis by promoting osteogenic differentiation of BMSCs via targeting *ICAM-1*^17^. Recently, researches showed that high glucose inhibited the osteogenic differentiation of BMSCs by regulating *miR-493-5p*/*ZEB2* axis^18^. Exactly, the roles and specific mechanism of *miR-493-5p* in osteoblast differentiation remain large elusive.

Autophagy maintains cytoplasmic balance by eliminating macromolecules and cells damaged by hunger or oxidative stress, which is beneficial to cell survival and growth^19^. The role of autophagy in reducing osteoporosis has been reported. Previous studies showed that in hypoxic microenvironments, autophagy maintained bone remodeling by removing reactive oxygen species, thereby alleviating osteoporosis^4^. In osteoporosis, oxidative stress induces TP53INP2 downregulation and suppresses osteogenic differentiation of BMSCs during osteoporosis through the autophagy degradation pathway^20^. Furthermore, autophagy is regulated by a variety of mechanisms, such as transcription factors, miRNA, and other related signaling pathways. Li et al. reported that *miR-223* inhibited autophagy by targeting *ATG16L1* to promote central nervous system inflammation^21^. Importantly, *miR-15b* accelerated the development of osteoporosis by regulating osteocyte differentiation and autophagy^22^. Moreover, *miR-493-5p* also had been described as a autophagy-related miRNA in bladder cancer^23^. However, it is not clear whether *miR-493-5p* affects autophagy and regulates the osteogenic differentiation of BMSCs and osteoporosis progression.

Strikingly, preliminary bioinformatics analysis found there was a putative binding target of *miR-493-5p* on the 3′-UTR regions of *Mef2c*. Previous literature revealed that single nucleotide polymorphism (SNP) of Mef2c gene locus was greatly correlated to adult osteoporosis and osteoporotic fractures^24^. More importantly, it also observed that enhancement of the p38/Mef2c pathway might be associated with autophagy activation^25^. Therefore, we hypothesized that *SNHG14* regulated the expression of *Mef2c* and activated autophagy through *miR-493-5p*, promoting osteogenic differentiation of BMSCs, thus alleviating osteoporosis development.

## Results

### SNHG14 gradually enhanced in osteogenic differentiation of BMSCs

**Fig. 1:**
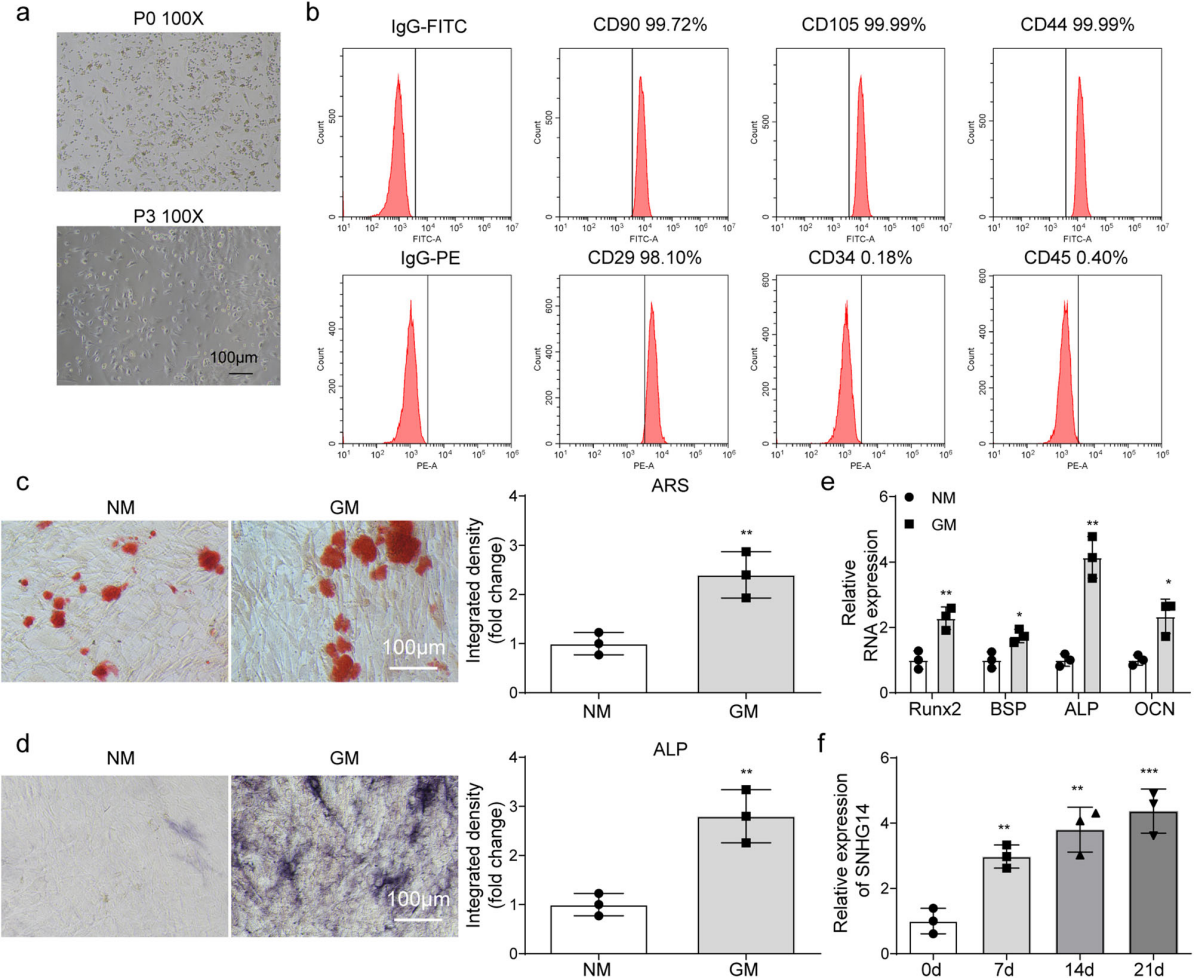
SNHG14 gradually increased in osteogenic differentiation of BMSCs. **a** Microscope observed the morphology of isolated BMSCs (100 ×). **b** Flow cytometry detected BMSCs surface antigen molecules, including CD90, CD105, CD44, CD29, CD34 and CD45. BMSCs were cultured in osteogenic medium for 21 days. **c** Alizarin Red S (ARS) staining analyzed mineralized nodules of BMSCs. **d** Representative images of ALP staining. **e**, **f** qRT-PCR assessed the level of *SNHG14* and osteogenic differentiation markers, including *Runx2*, *BSP*, *ALP,* and *OCN*. Data shown as Mean ± SD. **c**–**e** diagram using students’ *t*-test, and **f** diagram using one-way ANOVA with turkey post-test. NM indicates normal growth medium, GM indicates differentiation medium. *N* = 3, *p < 0.05, **p < 0.01, ***p < 0.001.

BMSCs were firstly isolated from mouse femur and tibia tissues. The results of microscopic morphological observation showed that the P0 generation cells were round or fusiform, and the P3 generation cells were fusiform. The adhesion of P0 generation cells was lower than that of P3 generation cells (Fig. 1a), which accorded with the morphological characteristics of BMSCs^26^. Flow cytometry analysis indicated that BMSCs surface antigen molecules CD90, CD105, CD44, and CD29 were positive, while CD34 and CD45 were negative (Fig. 1b), these findings suggested that BMSCs was isolated successfully. After the induction of osteogenic differentiation for 21 days, obvious mineralized nodules appeared in BMSCs (Fig. 1c). ALP staining analysis indicated that the ALP activity of BMSCs were significantly increased after the induction of osteogenic differentiation (Fig. 1d). Osteogenic differentiation markers levels, including *Runx2*, *BSP*, *ALP* and *OCN*, were strikingly increased in BMSCs following the induction of osteogenic differentiation (Fig. 1e). Next, *SNHG14* expression was gradually increased during BMSCs osteogenic differentiation (Fig. 1f). In short, these results showed BMSCs were successfully isolated from mice tissue.

### SNHG14 knockdown inhibited osteogenic differentiation of BMSCs

**Fig. 2:**
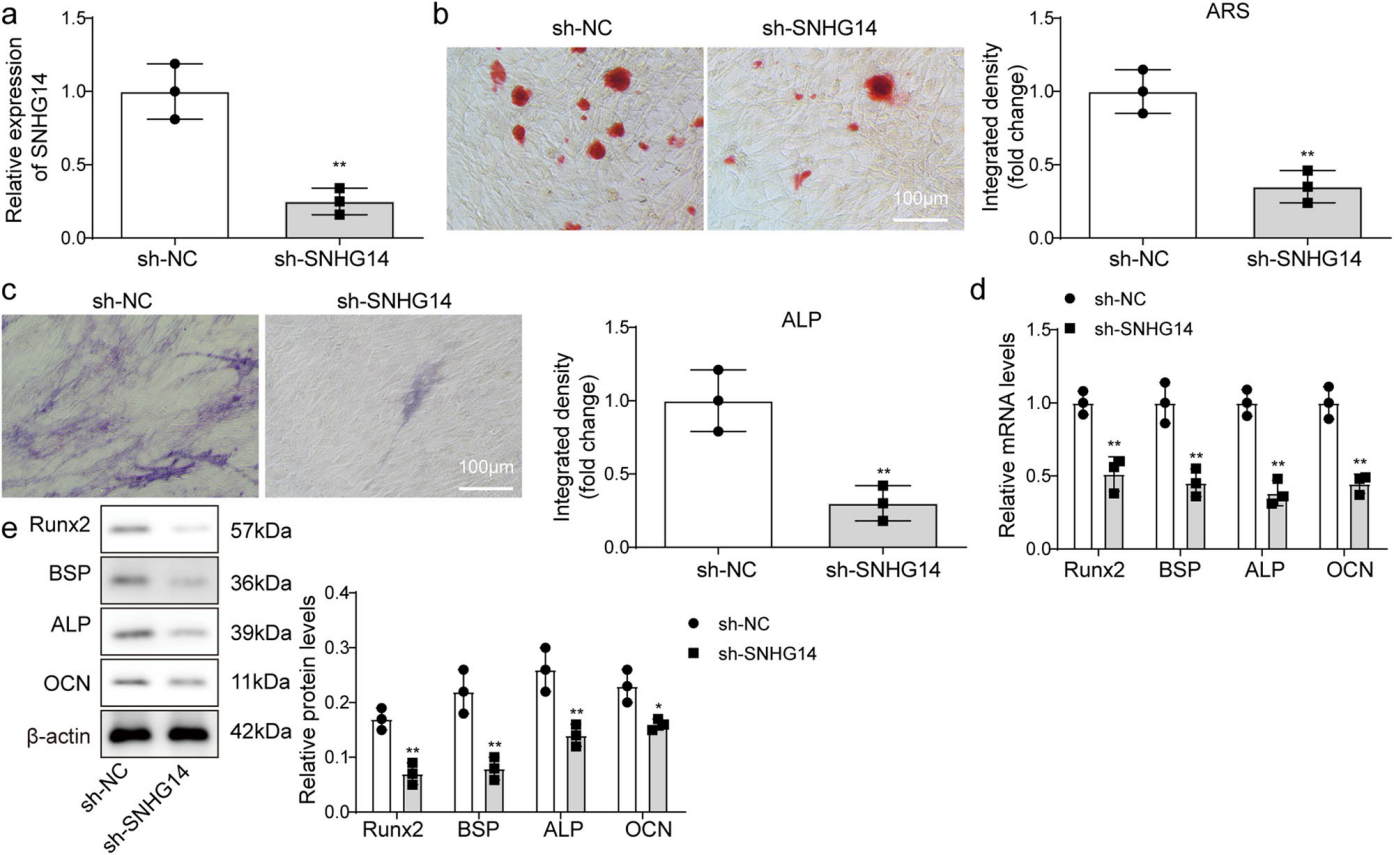
SNHG14 knockdown inhibited osteogenic differentiation of BMSCs. sh-SNHG14 was transfected into BMSCs for SNHG14 knockdown, sh-NC served as the negative control. 48 h later, sh-SNHG14 transfected BMSCs were further cultured in osteogenic differentiation medium for 21 days, sh-NC transfected BMSCs served as control. **a** qRT-PCR detected *SNHG14* mRNA level. **b** ARS staining analyzed the mineralized nodules of BMSCs. **c** Representative images of ALP staining. **d** The mRNA levels of *Runx2*, *BSP*, *ALP,* and *OCN* were assessed by qRT-PCR. **e** The protein levels of Runx2, BSP, ALP, and OCN were measured by Western blot. Data shown as Mean ± SD. Figure 2 using students’ *t*-test. *N* = 3, *p < 0.05, **p < 0.01, ***p < 0.001.

To investigate the effect of SNHG14 on osteogenic differentiation of BMSCs, sh-*SNHG14* was transfected into BMSCs for *SNHG14* knockdown, sh-NC served as the negative control. qRT-PCR analysis showed that *SNHG14* expression was significantly down-regulated in BMSCs after transfected with sh-*SNHG14* for 48 h (Fig. 2a). Subsequently, transfected BMSCs were further cultured in osteogenic differentiation medium. SNHG14 knockdown blocked the formation of BMSCs mineralized nodules (Fig. 2b). ALP staining analysis displayed that the ALP activity in BMSCs were decreased by SNHG14 knockdown (Fig. 2c). Moreover, qRT-PCR and western blot analysis disclosed that SNHG14 knockdown significantly inhibited the mRNA and protein level of Runx2, BSP, ALP and OCN (Fig. 2d, e). To sum up, SNHG14 knockdown inhibited osteogenic differentiation of BMSCs.

### SNHG14 targeted miR-493-5p to regulate Mef2c expression

**Fig. 3:**
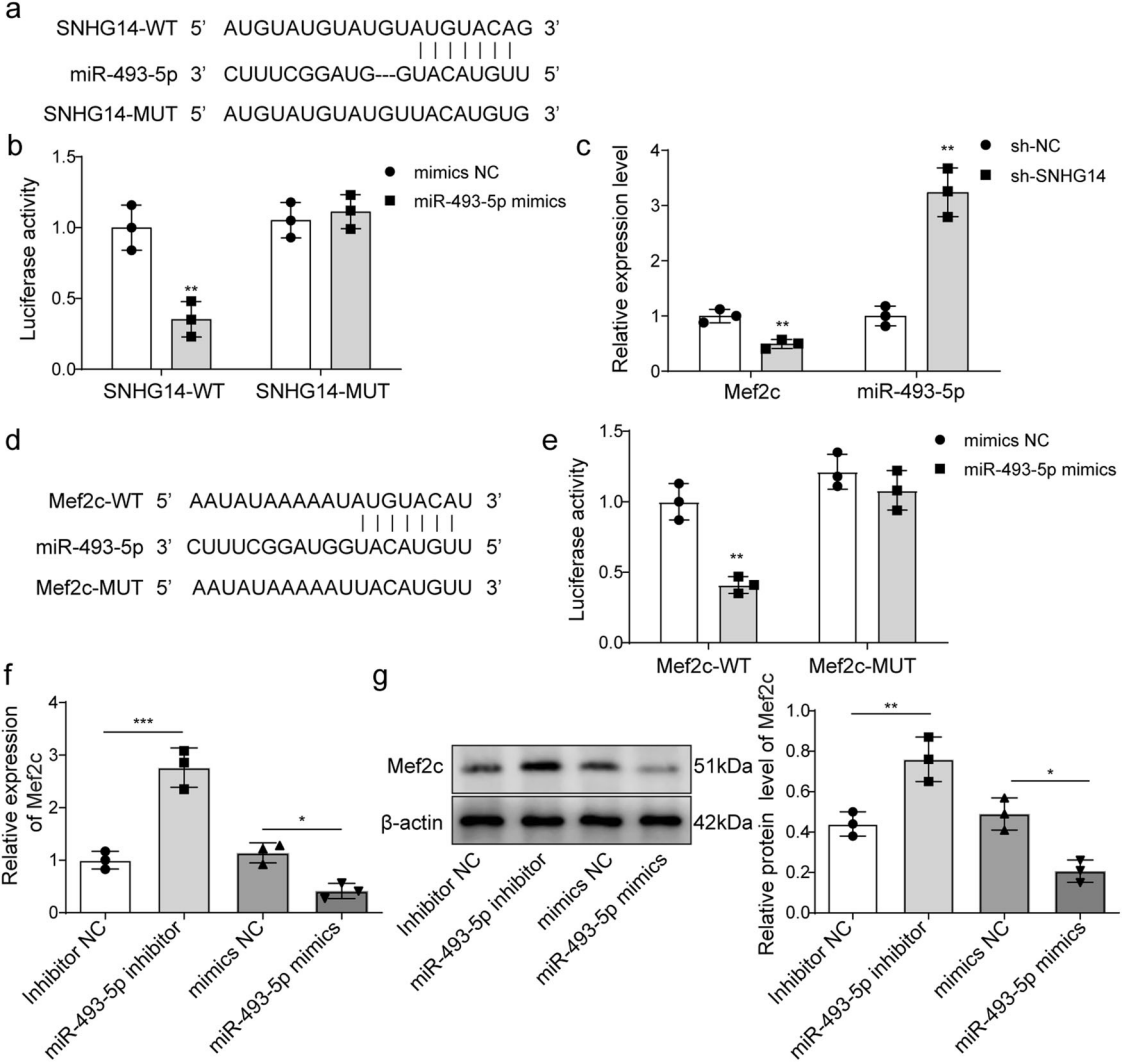
SNHG14 targeted miR-493-5p, and Mef2c served as a target of miR-493-5p. **a** miRDB software (http://www.mirdb.org/index.html) predicted the binding sites of SNHG14 and miR-493-5p. **b** SNHG14 and miR-493-5p binding relationship was confirmed by dual-luciferase reporter assay. sh-SNHG14 was transfected into BMSCs for SNHG14 knockdown, sh-NC served as control. **c** qRT-PCR detected the mRNA levels of miR-493-5p and Mef2c in BMSCs after transfected with sh-SNHG14. **d** miRDB database (http://www.mirdb.org/index.html) predicted the downstream targets of miR-493-5p and Mef2c. **e** miR-493-5p and Mef2c binding relationship was confirmed by double luciferase reporter assay. **f** qRT-PCR detected *Mef2c* level in BMSCs after transfected with miR-493-5p mimics or inhibitor. **g** Western blot measured Mef2c protein level in BMSCs after transfected with miR-493-5p mimics or inhibitor. Data shown as Mean ± SD. **b**, **c**, **e** diagram using students’ *t*-test, and **f**, **g** diagram using one-way ANOVA with turkey post-test. *N* = 3, *p < 0.05, **p < 0.01, ***p < 0.001.

MiRDB software (http://www.mirdb.org/index.html) was applied to search the downstream targets gene of SNHG14. As shown in Fig. 3a, a binding site between SNHG14 and miR-493-5p was found. Subsequently, the direct binding relationship between SNHG14 and miR-493-5p was confirmed by dual-luciferase reporter assay. The results showed that co-transfection of SNHG14-wt plasmid and miR-493-5p mimetic into BMSCs resulted in a decrease in luciferase activity, which was blocked when mutations occurred at the assumed binding site (Fig. 3b). *miR-493-5p* level was significantly up-regulated in BMSCs after transfected with sh-SNHG14. Inversely, *SNHG14* knockdown significantly decreased *Mef2c* level (Fig. 3c). Subsequently, miRDB software (http://www.mirdb.org/index.html) was used to search the downstream targets gene of miR-493-5p. Importantly, a binding site between miR-493-5p and 3′-UTR of Mef2c was found (Fig. 3d). And the luciferase activity of Mef2c-wt reported plasmids was inhibited by co-transfection of miR-493-5p mimics, but the co-transfection of miR-493-5p mimics did not affect the luciferase activity of Mef2c-mut reported plasmids (Fig. 3e). Furthermore, miR-493-5p inhibition significantly increased Mef2c expression, whereas Mef2c expression was significantly inhibited by miR-493-5p overexpression (Fig. 3f, g). Taken together, these data suggested that SNHG14 targeted miR-493-5p, and Mef2c served as a target of miR-493-5p.

### The inhibitory effect of miR-493-5p mimic on osteogenic differentiation was rescued by overexpressing SNHG14

**Fig. 4:**
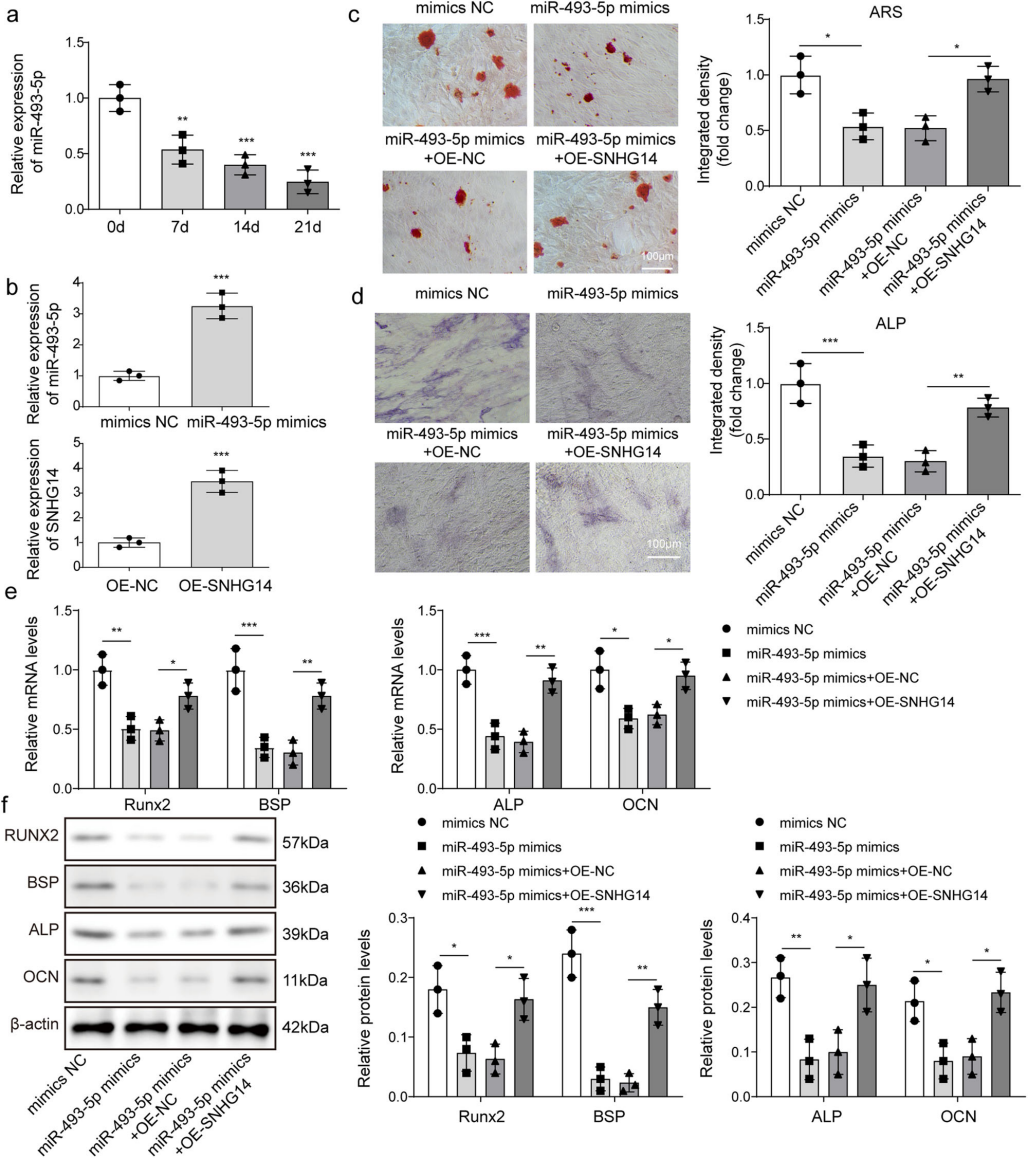
The inhibition of osteogenic differentiation of BMSCs induced by miR-493-5p mimics was relieved by SNHG14 overexpression. BMSCs were transfected with miR-493-5p mimics and OE-SNHG14 for the overexpression of miR-493-5p and SNHG14. NC mimics and OE-NC vector served as the negative control. **a** qRT-PCR detected miR-493-5p expression in BMSC by different time induced. **b** qRT-PCR detected *miR-493-5p* and *SNHG14* mRNA level. **c** ARS staining analyzed the mineralized nodules of BMSCs. **d** Representative images of ALP staining. **e** the mRNA levels of *Runx2*, *BSP*, *ALP,* and *OCN* were assessed by qRT-PCR. **f** the protein levels of Runx2, BSP, ALP, and OCN were measured by Western blot. Data shown as Mean ± SD. **b** diagram using students’ *t*-test, and **a**, **c**–**f** diagram using one-way ANOVA with turkey post-test. *N* = 3, *p < 0.05, **p < 0.01, ***p < 0.001.

First, we found that the expression of miR-493-5p was gradually reduced during osteoblast differentiation (Fig. 4a). Next, we investigated the role of SNHG14/miR-493-5p axis in osteogenic differentiation of BMSCs. The expression of *miR-493-5p* or *SNHG14* was significantly up-regulated in BMSCs after transfected with miR-493-5p mimics or OE-SNHG14 (Fig. 4b). Then, BMSCs overexpressed with miR-493-5p were further transfected with OE-SNHG14, and transfected BMSCs were further cultured in osteogenic differentiation medium. Alizarin Red S staining results indicated that miR-493-5p overexpression blocked the formation of BMSCs mineralized nodules, while SNHG14 overexpression reversed the effect of miR-493-5p overexpression (Fig. 4c). MiR-493-5p overexpression decreased ALP activity of BMSCs, whereas SNHG14 overexpression reversed the downward trend (Fig. 4d). Furtherly, the mRNA and protein level of osteogenic differentiation marker proteins, including Runx2, BSP, ALP and OCN were down-regulated by miR-493-5p overexpression, but the downward trend was reversed by SNHG14 overexpression (Fig. 4e, f). Overall, the inhibitory effect of miR-493-5p mimic on osteogenic differentiation was rescued by overexpressing SNHG14.

### Inhibition of miR-493-5p activated Mef2c-mediated autophagy to accelerate osteogenic differentiation of BMSCs

**Fig. 5:**
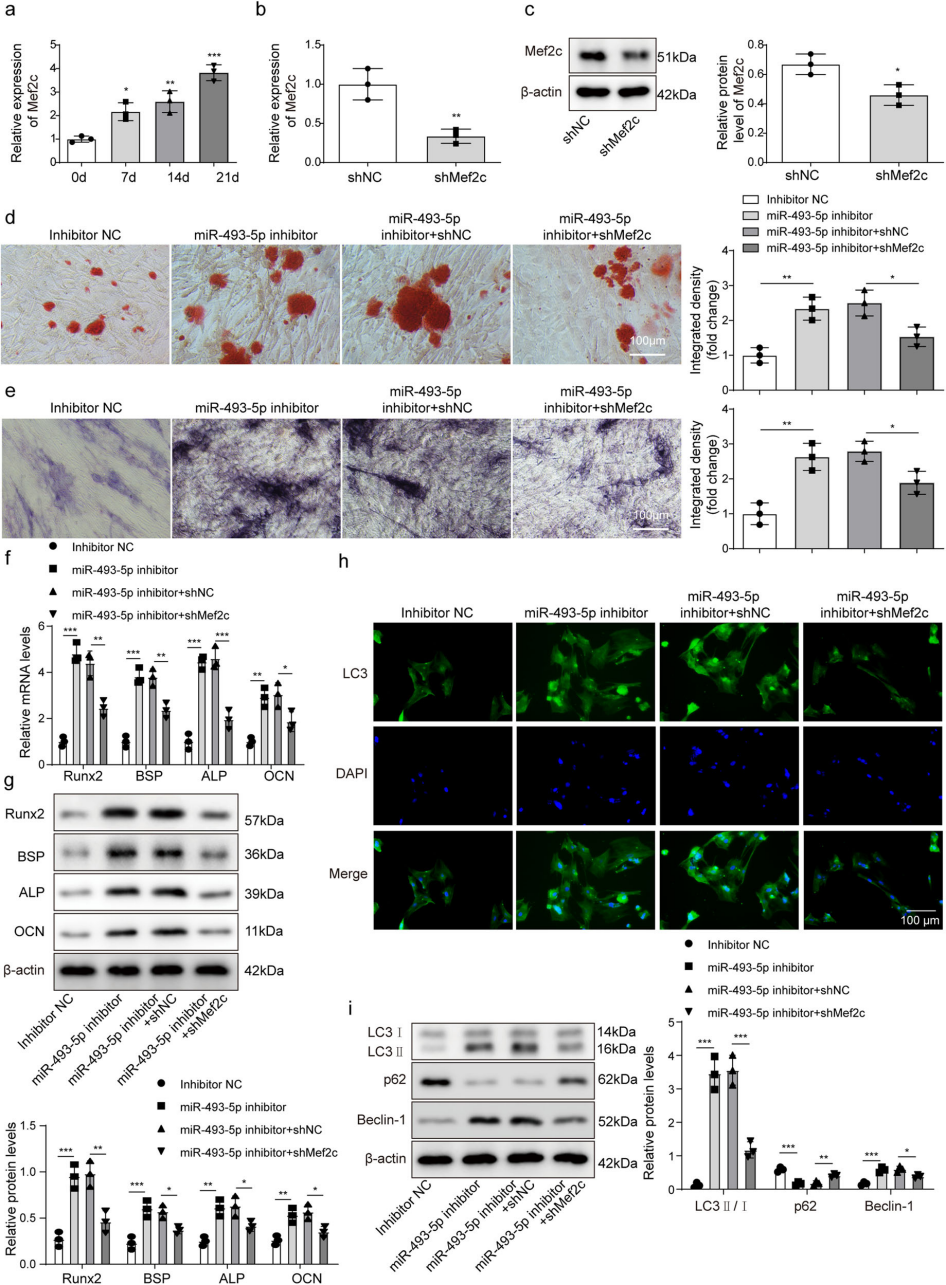
Inhibition of miR-493-5p activated Mef2c-mediated autophagy to accelerate osteogenic differentiation of BMSCs. **a** qRT-PCR detected level of Mef2c after osteogenic induction for 0, 7, 14, and 21 days. sh-Mef2c and sh-NC were transfected into BMSCs, respectively. sh-NC served as control. **b**, **c** qRT-PCR, and Western blot detected Mef2c expression. MiR-493-5p inhibitor or sh-Mef2c was co-transfected into BMSCs, and followed by exposed to osteogenic induction medium for 21 days. **d** ARS staining analyzed mineralized nodules of BMSCs. **e** Representative images of ALP staining. **f**, **g** the mRNA and protein levels of Runx2, BSP, ALP, and OCN were detected by qRT-PCR and Western blot. **h** Immunofluorescence assay detected the expression of LC3. **i** the expression of LC3II/I, Beclin-1 and p62 proteins were measured by Western blot. Data shown as Mean ± SD. **b**, **c** diagram using students’ *t*-test, and **a**, **d**–**i** diagram using one-way ANOVA with turkey post-test. *N* = 3, *p < 0.05, **p < 0.01, ***p < 0.001.

Based on previous finding^27^, we aimed to investigate whether Mef2c is related to the regulation of miR-493-5p in the differentiation of BMSCs. *Mef2c* expression was gradually increased in the process of osteogenic differentiation of BMSCs (Fig. 5a). Subsequently, we silenced Mef2c by transfecting sh-Mef2c into BMSCs, which was confirmed by RT-qPCR and western blot, showing Mef2c was reduced after sh-Mef2c transfection (Fig. 5b, c). Alizarin Red S and ALP staining demonstrated that miR-493-5p inhibition accelerated the formation of BMSCs mineralized nodules and ALP activity, but these effects were all abolished after Mef2c silencing (Fig. 5d, e). The high levels of osteogenic differentiation markers including Runx2, BSP, ALP, and OCN induced by miR-493-5p inhibitor were remarkably reversed by Mef2c knockdown (Fig. 5f, g). These findings suggested that miR-493-5p regulated osteogenic differentiation of BMSCs by targeting Mef2c. It was reported that autophagy was vital for the survival, differentiation, and function of osteocytes^28^. P38/Mef2c signaling was also identified to be linked to autophagy activation^25^. Herein, we further examined the expression of autophagy-related proteins during osteogenic differentiation of BMSCs. Immunofluorescence detected LC3 expression, and the results showed miR-493-5p inhibitor increased LC3 level, while Mef2c silencing revised the effect of miR-493-5p inhibitor (Fig. 5h). The results demonstrated that miR-493-5p inhibition enhanced the ratio of LC3II/I and Beclin-1 expression, but inhibited p62 expression, while these trends were strikingly reversed by Mef2c inhibition (Fig. 5i), suggesting the miR-493-5p/Mef2c axis participates in the osteogenic differentiation process of BMSC by regulating autophagy.

### SNHG14 overexpression activated Mef2c-mediated autophagy to accelerate osteogenic differentiation of BMSCs

We furtherly explore whether SNHG14 regulated osteogenic differentiation of BMSCs by regulating Mef2c-mediated autophagy. Alizarin Red S and ALP staining demonstrated that SNHG14 overexpression accelerated the formation of BMSCs mineralized nodules and ALP activity, but these effects were all abolished after Mef2c silencing (Supplementary Fig. 1a, b). The high levels of osteogenic differentiation markers including Runx2, BSP, ALP, and OCN induced by SNHG14 overexpression were remarkably reversed by Mef2c knockdown (Supplementary Fig. 1c, d). Besides, the level of LC3 in BMSCs was detected by immunofluorescence assay. Results indicated that SNHG14 overexpression enhanced LC3 expression, while this trend was strikingly reversed by Mef2c inhibition (Supplementary Fig. 1e). Western blot analysis also discovered that SNHG14 overexpression significantly elevated the levels of autophagy-associated proteins, including LC3II/I and Beclin-1, but reduced p62 level, while Mef2c knockdown reversed these trends (Supplementary Fig. 1f). These findings indicated that SNHG14 overexpression activated Mef2c-mediated autophagy to accelerate osteogenic differentiation of BMSCs.

### SNHG14 overexpression promoted bone formation and alleviated osteoporosis in vivo

**Fig. 6:**
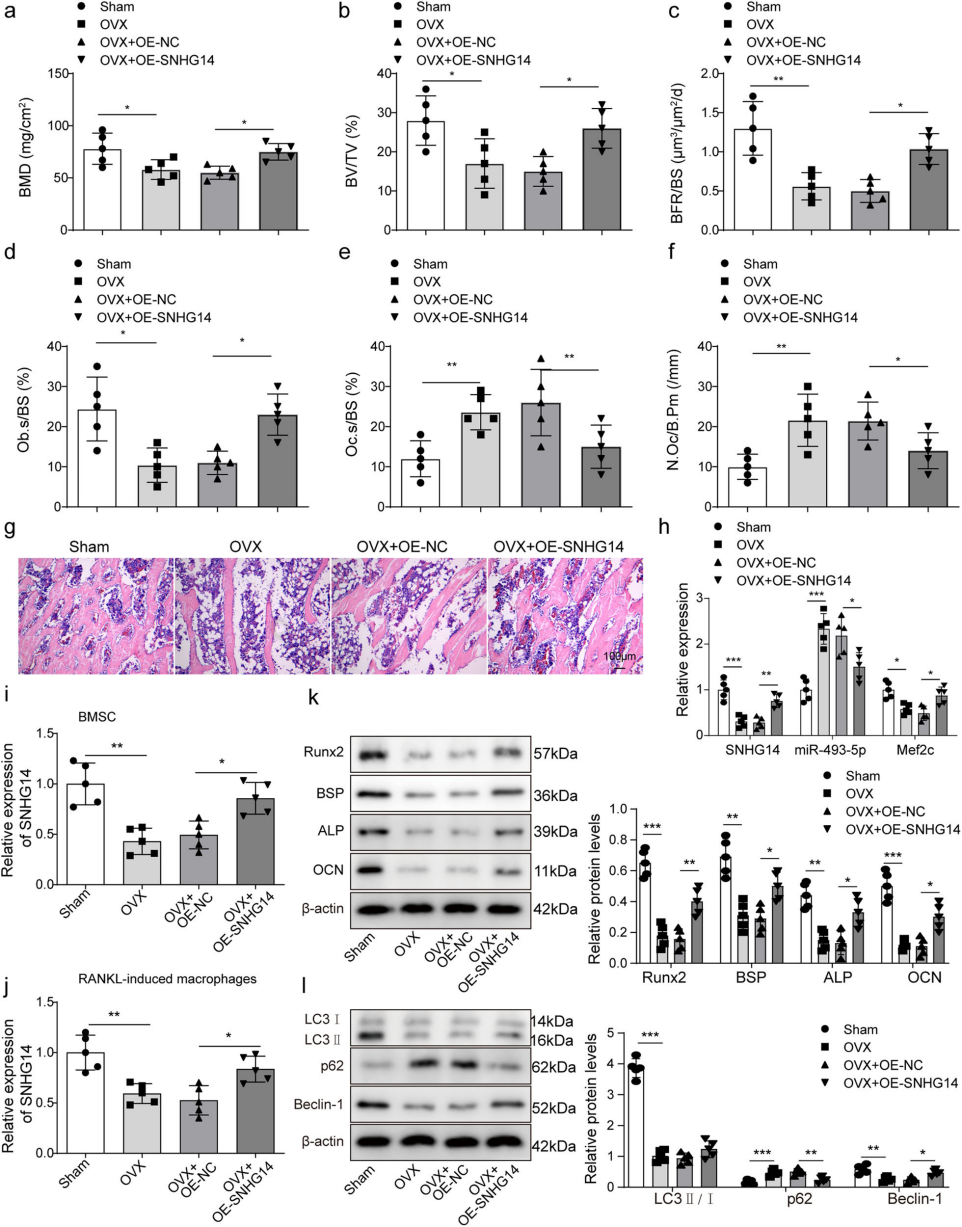
SNHG14 overexpression promoted bone formation and alleviated osteoporosis in vivo. The mice were randomly divided into 4 groups (*n* = 5/each group): Shame group, OVX group, OVX + OE-NC, OVX + OE-SNHG14 group. **a** PIXImus densitometer was used to measure BMD of femurs. **b** Micro-CT measured BV/TV of structural parameters. **c** Bone formation parameter (BFR/BS) in distal mouse femur. **d** Osteoblast parameter (Ob.S/BS) was measured by histomorphometric analysis. **e**, **f** Bone resorption parameters (Oc.S/BS and N.Oc/BS) in distal mouse femur. **g** H&E staining was used to detect the pathological change. **h** qRT-PCR assessed the levels of *SNHG14*, *miR-493-5p* and *Mef2c* genes. **i**, **j** qRT-PCR measured the expression of SNHG14 in BMSCs and osteoclast. **k** Western blot detected the levels of Runx2, BSP, ALP, and OCN. **l** Western blot measured the proteins levels of LC3II/I, Beclin-1, and p62. Data shown as Mean ± SD. Figure 6 using one-way ANOVA with turkey post-test. *N* = 5, *p < 0.05, **p < 0.01, ***p < 0.001.

For further explore the role of SNHG14 in bone formation in vivo, the osteoporotic mouse model. A statistically significant decrease in BMD and bone volume/total volume ratio (BV/TV) of OVX mice was detected, but OE-SNHG14 impeded the trends (Fig. 6a, b). Quantitative analysis of tissue morphology showed that BFR/BS, Ob.S/BS, Oc.S/BS, and N.Oc/B.Pm of OVX mice were all statistically increased compared to sham group, after treatment by OE-SNHG14, the above trends were all alleviated (Fig. 6c–f). Similarly, H&E staining showed that the number of bone trabeculae decreased in OVX mice compared with sham mice, but OE-SNHG14 reversed this trend (Fig. 6g). qRT-PCR analysis indicated that *SNHG14* and *Mef2c* expression was decreased, but *miR-493-5p* level was up-regulated in femurs and tibias tissues of OVX mice, while these effects were reversed by OE-SNHG14 treatment (Fig. 6h). Subsequently, BMSCs and osteoclasts were isolated from mouse bone marrow. qRT-PCR was performed to detected the level of SNHG14 in BMSCs and osteoclasts. Results discovered that compared to the sham group, the level of SNHG14 was significantly reduced after OVX treatment, while overexpression of SNHG14 had the opposite effects (Fig. 6i, j). Besides, Western blot analysis also discovered that the levels of osteogenic differentiation markers, including Runx2, BSP, ALP, and OCN, and autophagy-associated proteins, including LC3II/I and Beclin-1 were significantly elevated, but p62 level was reduced by OE-SNHG14 treatment compared to OVX group (Fig. 6k, l). These findings suggested that SNHG14 overexpression promoted bone information and alleviated osteoporosis, which might be correlated to Mef2c-mediated autophagy activation.

## Discussion

With the increase of the elderly population, osteoporosis has become a worldwide disease, which will increase the risk of fracture and affect human health. The decrease of osteogenic differentiation of BMSCs leads to the decrease of bone formation and accompanied by osteoporosis^29^. In the present work, we isolated BMSCs from femurs and tibias tissues of mice to establish an osteogenic differentiation model in vitro. Moreover, we also conducted an osteoporotic mouse model in vivo. The finally data showed that SNHG14 targeted miR-493-5p/Mef2c axis to regulate autophagy activation, resulting the promotion effect in osteogenic differentiation of BMSCs, suggesting SNHG14 might be an effective target for osteoporosis treatment.

LncRNAs are a type of universal genes with multiple biological functions. In this paper, our findings suggest that SNHG14 was a sponge of miR-493-5p. There is growing evidence that the lncRNA/miRNA axis is involved in regulating various physiological and pathological processes in BMSCs, including osteogenic differentiation. Liu et al. showed that SNHG14 promoted the osteogenic differentiation of BMSCs by regulating the miR-185-5p/WISP2 axis^30^. Similarly, our findings indicated that SNHG14 knockdown inhibited osteogenic differentiation of BMSCs. Mechanically, we found that there was a binding site between SNHG14 and miR-493-5p.

MiRNAs have been shown to be widely involved in various biological processes. In recent years, the abnormal expression of miRNAs in osteoporosis has received widespread attention^11^. As previous mentioned, kynurenine accumulation led to an oxidative stress environment, resulting in miRNA profile changes in BMSCs, including the increased let-7f-5p, miR-493-5p, and the decreased miR-1281, miR-330-3p, miR-210-3p, which were the key factor to modulate BMSCs proliferation and differentiation^31^. Importantly, it reported that high glucose regulated the osteogenic differentiation of BMSCs by regulating miR-493-5p^18^. In our present work, we have found that miR-493-5p was up-regulated in osteoporotic mice and reduced gradually during BMSCs osteogenic differentiation. Subsequently, we also observed that miR-493-5p functioned as a negative regulator of osteoblast differentiation of BMSCs to decrease the formation of mineralized nodules, ALP activity, and osteogenic markers, indicating miR-493-5p plays a negative role in osteogenic differentiation of BMSCs. What’s more, we found that miR-493-5p served as a target of SNHG14, and the inhibition of osteogenic differentiation of BMSCs induced by miR-493-5p overexpression was relieved by SNHG14 overexpression. These findings suggested that SNHG14 via targeting miR-493-5p to regulate osteogenic differentiation of BMSCs. Furthermore, SNHG14 participates in the regulation of autophagy through targeted regulation of miRNAs. Deng et al. reported that SNHG14 regulates mouse hippocampal neurons through miR-182-5p/BINP3 axis^32^. SNHG14 regulated autophagy through the miR-223-3p/Foxo3a axis to alleviate acute lung injury induced by lipopolysaccharide^33^. Notably, we also observed the autophagy activation in miR-493-5p silenced BMSCs, presenting the increase of LC3II/I, Beclin-1, and the reduction of p62. Similarly, Moreover, a great number of evidences was widely confirmed that autophagy activation was a critical driven factor in osteogenic differentiation^34–36^. Thus, it could be inferred that the anti-osteogenic differentiation of miR-493-5p might be correlated to the inactivation of autophagy, which deserves further verification in the future.

Mef2c is a transcription factor associated with various cells’ development, including bone cells^37^. However, the biological role of Mef2c in osteoporosis and related bone formation is multidirectional. On the hand, it has been showed that the double deficiency of Mef2c and Mef2d will lead to cartilage angiogenesis and ossification in mice^38^. Likewise, Takayuki et al. revealed that Mef2c play as a positive regulator of osteoclast differentiation by facilitating c-FOS and NFATc1^37^. On the other hand, Mef2c was negatively targeted by miR-23 in hBMSCs, and exhibited a promoting role in osteogenic differentiation by activating MAPK signaling^39^. In addition, Ni et al. investigation observed that enforced Mef2c expression could enhance p38, Osx and Runx2 and multiple osteogenesis‑associated genes in mouse MSCs^40^. From these observations, we believe that more experiments are needed to verify the exact biological functions and mechanisms of Mef2c. In this work, Mef2c was found gradually up-regulated during osteogenic induction of BMSCs, and the depletion of Mef2c led to an inhibitory role in osteogenic differentiation of BMSCs, which strikingly diminished miR-493-5p knockdown. Studies have shown that Mef2c is involved in the regulation of autophagy. For example, rapamycin treatment enhanced Mef2c expression, suggesting autistic behavior in rats can be improved by enhancing autophagy-activated p38/Mef2c signal pathway^25^. Importantly, we also observed that Mef2c silencing inhibited autophagy-related LC3II/I and Beclin-1 expression, but increased p62 accumulation, which consistent to Luo et al. findings^25^. More importantly, we have confirmed the targeting relationship between miR-493-5p and Mef2c. Therefore, we considered that Mef2c functioned as a promoting factor in osteogenic differentiation to involve in the regulation of miR-493-5p on BMSCs differentiation.

In summary, our findings firstly elucidated the roles and exact mechanism of SNHG14/miR-493-5p/Mef2c axis in osteogenic differentiation of BMSCs. It has demonstrated that SNHG14 by regulating miR-493-5p/Mef2c-mediated autophagy activation, leading to the promotion effect on osteogenic differentiation of BMSCs, thereby improving osteoporosis progression. In conclusion, SNHG14 activated autophagy via regulating miR-493-5p/Mef2c axis to alleviate osteoporosis progression. Our research provided more evidence for further understanding of the pathological mechanism of osteoporosis.

## Materials and methods

### Isolation and identification of BMSCs

Balb/c female mice were purchased from CasGene Biotech company (Beijing, China, 20 g to 25 g). The 8-week-old mice were sacrificed according to Animal Care and Use Guidelines of the First Affiliated Hospital, Department of Spine Surgery, Hengyang Medical School, University of South China (NO. 2021ll0518003). In detail, isoflurane anesthetized mice were subjected to 15 min in CO_2_ and were euthanized. Subsequently, the femur and tibia of mice were immediately removed for BMSCs isolation, according to previously description^5^. BMSCs cells were washed with syringe needle system and cultured in DMEM medium (Invitrogen, Carlsbad, CA, USA) containing 10% FBS (Solarbio, Beijing, China). For osteogenesis, BMSCs (4 × 10^5^ cells/cm^2^) were cultured in α-MEM plus 10% FBS added with 50 μg/mL ascorbate-2, 10 mM β-glycerol phosphate and 0.1 μM dexamethasone for 21 d. Finally, the morphology of BMSCs was observed by microscope.

### Flow cytometric analysis of BMSCs

The BMSCs cells were isolated with trypsin and washed twice with PBS. The cell suspension containing 1 × 10^5^ cells was fixed in precooled 2% frozen formaldehyde. Subsequently, BMSCs were incubated with anti-CD90 (Abcam, ab3105, 1:50), anti-CD105 (Abcam, ab2529, 1:50), anti-CD44 (Abcam, ab112178, 1:100), anti-CD29 (Abcam, ab36219, 1:50), anti-CD34 (Abcam, ab81289, 1: 50) and anti-CD45 (Abcam, ab10558, 1:50) for 30 min. Finally, FACS Calibur system was used for flow cytometry, and cellquest graphics software was used to collect and analyze data.

### Alizarin red S staining

After osteogenic induction of BMSCs, calcification was analyzed by Alizarin Red S staining, according to previous reports^41^. In detail, PBS was used to wash the BMSCs cultured in osteogenic differentiation medium for 15 days. Subsequently, BMSCs was fixed for 30 min with 10% formalin. After washing with PBS for 3 times, the cells were stained with alizarin red for 3 h at room temperature. Finally, wash the BMSCs with distilled water and take pictures under a microscope (OLYMPUS, Tokyo, Japan).

### ALP staining

ALP staining was performed according to previous reports^42^. BMSCs was fixed with 4% formaldehyde, incubated with BCIP/NBT solution (Beyotime, Nanjing, China) under dark conditions, and washed with PBS three times to stop the reaction. Subsequently, the coloring was observed with a microscope, and the results were statistically analyzed.

### BMSCs transfection

The shRNAs targeting *SNHG14* or Mef2c, *miR-493-5p* inhibitor, *miR-493-5p* mimics, and their negative control (shNC, inhibitor NC, mimics NC) were synthesized by GeneChem (Shanghai, China). And cells were transfected with above plasmids by liposome 2000 reagent (Invitrogen, CA, USA). Then, lentiviral carried with overexpression vectors (OE-SNHG14) were co-transfected into human embryonic kidney 293 T (HEK293T) cells, and the three packaging plasmids included pHBLVTM, psPAX2, and pMD2.G. Lentiviral particles were obtained after 48 h of transfection via cell harvest and concentration with ultracentrifugation at 72,000 *g* for 2 h. Following titer determination, BMSCs were transfected at a multiplicity of infection (MOI) of 50 for 24 h.

### qRT-PCR

The total RNA of the femur was isolated using an EASYspin Plus Bone Tissue RNA Kit (RN54, Aidlab Biotechnologies Co., Ltd., Beijing, China) according to the manufacturer’s instructions. The concentration and purity of the total RNA was calculated by measuring the absorbance at 260 nm and 280 nm. Total RNA was extracted from cells using a High Pure RNA isolation kit (BioTeke, China). cDNA was reversely transcribed from total RNA using M-MLV reverse transcriptase and random primers (Invitrogen, CA, USA). miR-493-5p level was detected by Taqman microRNA assay kit (Takara). Data analysis used U6 as endogenous control. A one-step SYBR Prime Script Plus RT-PCR kit (Takara) detected *SNHG14*, *OCN*, *ALP*, *BSP*, *Runx2,* and *Mef2c* levels. Standard procedure for two-step PCR amplification: stage 1, pre-denaturation, 95 °C 30 s. Stage 2, PCR reaction, 95 °C 5 s, 60 °C 30 s (40 cycles). Finally, extend 72 °C for 5 min. GAPDH served as endogenous control. The 2^−ΔΔCt^ method calculated the relative expression level of genes. Primers were shown in Table 1.

**Table 1:** Primer sequences.

Primer name	Primer sequences (5′-3′)
F-SNHG14	GGGTGTTTACGTAGACCAGAACC
R-SNHG14	CTTCCAAAAGCCTTCTGCCTTAG
F-miR-493-5p	GCCGAGTTGTACATGGTAGG
R-miR-493-5p	CTCAACTGGTGTCGTGGA
F-Mef2c	ATCCCGATGCAGACGATTCAG
R-Mef2c	AGATCTGACATCCGGTGCAG
F-Runx2	TCTTGTTCAGGTTACCAGGT
R-Runx2	GGACCGTCCACTGTCACTTT
F-BSP	CGGCGATAGTTCCGAAGAGG
R-BSP	GTTGGAGTGCCGCTAACTCA
F-ALP	AACCCAGACACAAGCATTCC
R-ALP	CCAGCAAGAAGAAGCCTTTG
F-OCN	GAGACACCACCCCCTGTAAA
R-OCN	GAGACACCACCCCCTGTAAA
F-U6	CTCGCTTCGGCAGCACA
R-U6	AACGCTTCACGAATTTGCGT
F-GAPDH	AGCCCAAGATGCCCTTCAGT
R-GAPDH	CCGTGTTCCTACCCCCAATG

### Western blot

After the femurs were crushed in liquid nitrogen, then bone tissue protein extraction kit (BestBio, Shanghai, China) was used to extract the protein in bone tissues. BMSCs were gently lysed by incubation in ice-cold RIPA buffer (Beyotime, China) containing protease inhibitors (Beyotime, China) for 30 min followed by centrifugation at 12,000 × *g* for 15 min; then, the supernatant was collected. The total protein concentration was determined using a BCA kit (Beyotime, Nanjing, China). Protein (20 μg) was separated by 10% sodium dodecyl sulfate-polyacrylamide gel electrophoresis and electroprinted on polyvinylidene fluoride membranes produced by Invitrogen in California, USA. The PVDF membrane was incubated with anti-OCN (Abcam, ab93876, 1:1000), anti-ALP (Abcam, ab229126, 1:1000), anti-BSP (GeneTex, GTX12155, 1:2000), anti-RUNX2 (Abcam, ab236639, 1:1000), anti-Mef2c (Abcam, ab211493, 1:1000), anti-LC3 (Abcam, ab128025, 1:1000), anti-p62 (Abcam, ab109012, 1:10000) and anti-Beclin-1 (Abcam, ab207612, 1:2000) overnight at 4 °C. Subsequently, the second strain of anti-rabbit IgG antibody was conjugated to horseradish peroxidase (Abcam, ab150077, 1:2000) on the membrane for 2 h at room temperature, and the bands were detected with an immobilized Western chemiluminescence meter (AutoLumo A2000Plus, Autobio, Zhengzhou, China). Finally, the strength of the bands was quantified using ImageJ software. Anti-β-actin (Abcam, ab8226, 1:1000) acted as internal reference. All western blot images have been cropped according to the protein molecular weight recommended by the manufacturer’s instructions.

### Dual-luciferase reporter assay

miRDB software (http://www.mirdb.org/index.html) to predict the potential binding site between SNHG14, miR-493-5p and Mef2c 3′-UTR. Subsequently, the physical interaction between SNHG14, miR-493-5p, and Mef2c was confirmed by dual-luciferase reporter assay. In detail, the wild type (wt) or mutation type (mut) 3′-UTR fragments of *Mef2c* or *SNHG14* were amplified by PCR and followed by inserted into pmirGLO vector (Promega, Fitchburg, WI, USA) to construct recombinant luciferase vector (Mef2c-wt and Mef2c-mut). The *Mef2c*-wt/mut or *SNHG14*-wt/mut vectors were co-transfected with miR-493-5p mimics or mimics NC into BMSCs by lipofectamine 2000 reagent (Invitrogen, CA, USA). Luciferase activity was assessed 48 h after transfection.

### Osteoporotic mouse model

CasGene Biotech company (Beijing, China, 20 g to 25 g) provided 8-week-old Balb/c female mice. The mice were kept in standard cages with a room temperature of about 25 °C, a humidity of about 60%, a light-dark cycle of 12 h, and adequate food and water every day. All experimental produce was approved by the Ethics Committee of the First Affiliated Hospital, Department of Spine Surgery, Hengyang Medical School, University of South China (NO. 2021ll0518003). The mice were randomly divided into 4 groups (*n* = 5/each group): Sham group, OVX group, OVX + vector, OVX + Overexpressed (OE)-SNHG14. After anesthesia with pentobarbital sodium (50 mg/kg, intraperitoneal injection), the mice were subjected to sham operation or surgical ovariectomy (OVX), as described earlier^43^. Ovariectomy was performed in the OVX group. The bilateral ovaries of the sham group were identified and placed back into the abdominal cavity. Two months after ovariectomy, OVX mice were tail vein injected with lentivirus-packaged OE-NC or lentivirus-packaged OE-SNHG14 (10 nmol/per mouse). Once a week, four weeks after continuous injection, the mouse was euthanized by using CO_2_.

### Micro-CT analysis

Microcomputed tomography (micro-CT) was applied to determine bone mass. After anesthesia with 1% pentobarbital, the mice were secured on the table in the prone position. Then, femurs were imaged by microcomputed tomography (PerkinElmer, USA) at 55 kV at an 8 μm voxel size. After the image was reconstructed, the area of interest (ROI) of trabecular bone was analyzed within the 0.3–0.8 mm distal metaphysis from the growth plate. Analyze V12.0 software was used to analyze the data and quantify the parameters, including bone mineral density (BMD), trabecular bone thickness (Tb. Th), trabecular bone number (Tb. N), bone volume per tissue volume (BV/TV), and trabecular separation (Tb. Sp).

### Osteoclast culture

BMSCs were harvested from the femora and tibiae of 8-week-old mice. Then, the bone marrow cells (0.5 × 10^6^) were differentiated into bone marrow-derived macrophages in α-MEM supplemented with 50 ng/mL recombination macrophage colony stimulating factor Glutamax, 10% heat-inactivated FBS, and 1% PBS in a 12-well plate for 16 h in a 37 °C incubator with 5% CO_2_. The non-adherent cells were then maintained in the presence of 50 ng/mL receptor activator for nuclear factor-κB ligand (RANKL) and 50 ng/mL M-CSF for an additional 5 days. For the coculture assay, osteoclasts were cocultured with BMSCs at a 1:3 ratio for 5 d in a Transwell system. Tartrate resistant acid phosphatase (TRAP) staining was performed using a kit (Solarbio, China) following the manufacturer’s protocols.

### Histomorphometric analysis

Balb/c female mice were intraperitoneally injected with 20 mg/kg calcein (Sigma–Aldrich, USA), dissolved in PBS at a concentration of 2 mg/ml with 1 mg/ml NaHCO3 (Sigma–Aldrich, USA) at 16 d and 2 d before sacrifice. After sacrifice, the left femur was obtained, fixed in 4% formaldehyde, and embedded in methyl methacrylate. The specimen was cut into 30 mm slices by using a microtome (SP1600; Leica, Germany) under the condition of avoiding light. Then, cortical endosteum surfaces were evaluated using a fluorescence microscope (STP6000; Leica, Germany) with an excitation wavelength of 488 nm. Bone formation rate/bone surface area (BFR/BS), osteoblast surface area/osteoblast surface area (Ob.S/BS), osteoclast surface /bone surface area (Oc. S/BS) and number of osteoclast per bone surface (N.Oc/B.Pm) were used for quantitative analysis using ImageJ 1.47 software.

### Hematoxylin-eosin (H&E) staining

The femur was decalcified by 10% EDTA and then embedded in paraffin. Five-micrometer sagittal sections of the metaphysis were prepared, stained with Toluidine blue, TRAP, hematoxylin-eosin (HE), and observed by light microscopy (Zeiss, Germany).

### Statistics and reproducibility

Mean ± standard deviation (SD) represents data from at least three independent trials. All data were statistically analyzed using GraphPad Prism 5.0 Software (GraphPad Software, Inc.). Student’s *t*-test was used for comparison between the two groups, and One-way ANOVA with turkey post-test were used for multi-group comparison. The difference between the two groups was statistically significant (*P* < 0.05).

### Reporting summary

Further information on research design is available in the Nature Portfolio Reporting Summary linked to this article.

### Supplementary information

Supplementary Information

Description of Supplementary Materials

Supplementary data1

Reporting Summary

## Data Availability

All data that have been generated or analyzed during this study are included in the published article and the associated supplementary files. Where cropped western blot images have been used in the results figures, uncropped images have also been provided (Supplementary Fig. 7); raw data for graphs are presented in Supplementary Data l.
