# Supplementary material for: LncRNA SNHG14 activates autophagy via regulating miR-493-5p/Mef2c axis to alleviate osteoporosis progression
Source: Commun Biol. 2023 Nov 4;6:1120. doi: 10.1038/s42003-023-05493-8 (PMC10625635; doi:10.1038/s42003-023-05493-8)
Supplement: Supplementary file 1 — Supplementary Information [file 42003_2023_5493_MOESM1_ESM.pdf]

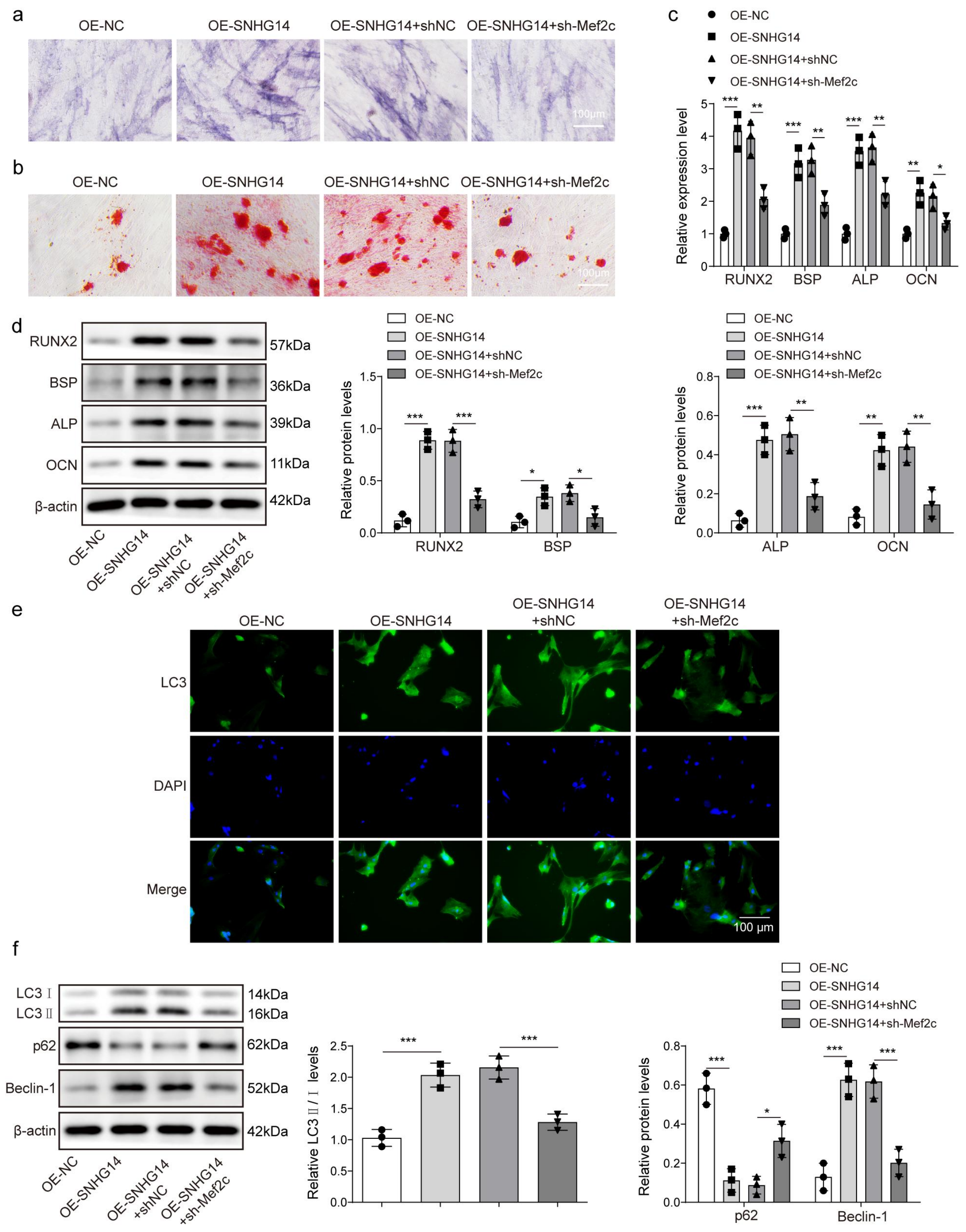

Supplementary Figure 1 SNHG14 overexpression activated Mef2c-mediated autophagy to accelerate osteogenic differentiation of BMSCs.

OE-SNHG14 and sh-Mef2c were transfected into BMSCs. **a**, ARS staining analyzed the mineralized nodules of BMSCs. **b**, Representative images of ALP staining. **c**, the

mRNA levels of Runx2, BSP, ALP and OCN were assessed by qRT-PCR. **d**, the protein levels of Runx2, BSP, ALP and OCN were measured by Western blot. **e**, the level of LC3 in BMSCs was detected by immunofluorescence assay. **f**, the expression of LC3II/I, Beclin-1 and p62 proteins were measured by Western blot. Data shown as Mean ± SD. Figure 6 using one-way ANOVA with turkey post-test. N=3, *p<0.05, **p<0.01, ***p<0.001.

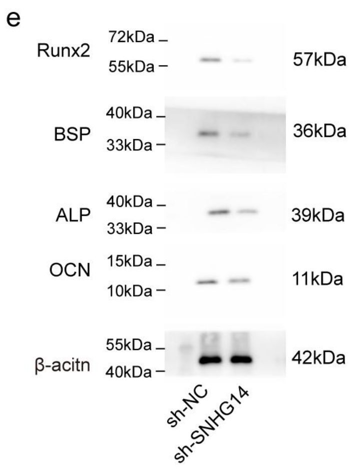

Supplementary Figure 2. Uncropped and unedited western blot images for Fig. 2.

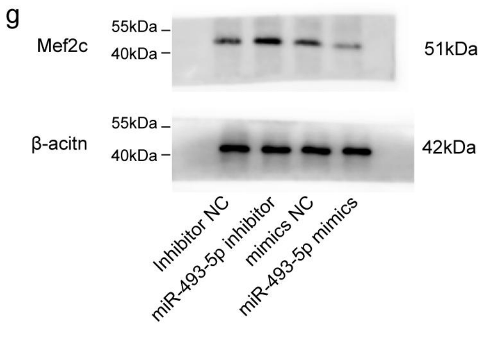

Supplementary Figure 3. Uncropped and unedited western blot images for Fig. 3.

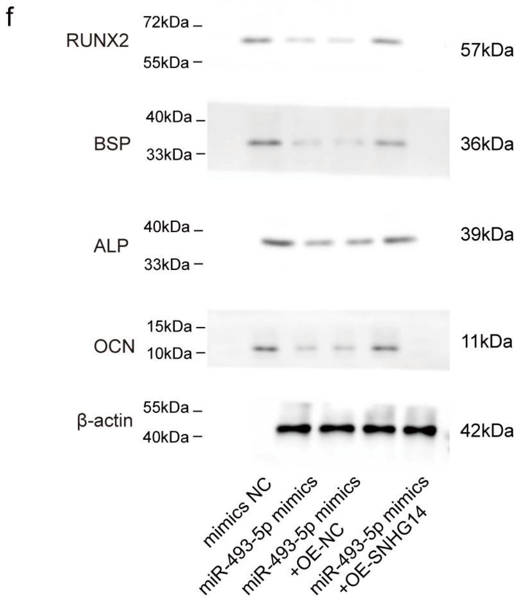

Supplementary Figure 4. Uncropped and unedited western blot images for Fig. 4.

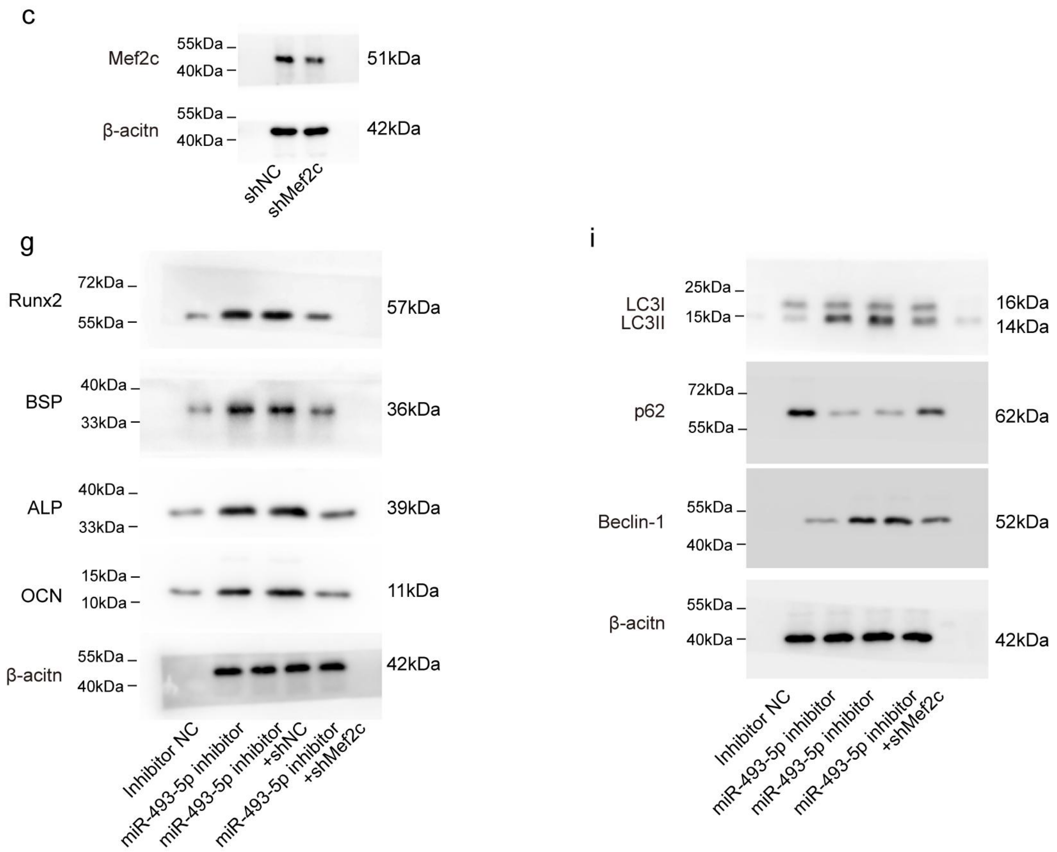

Supplementary Figure 5. Uncropped and unedited western blot images for Fig. 5.

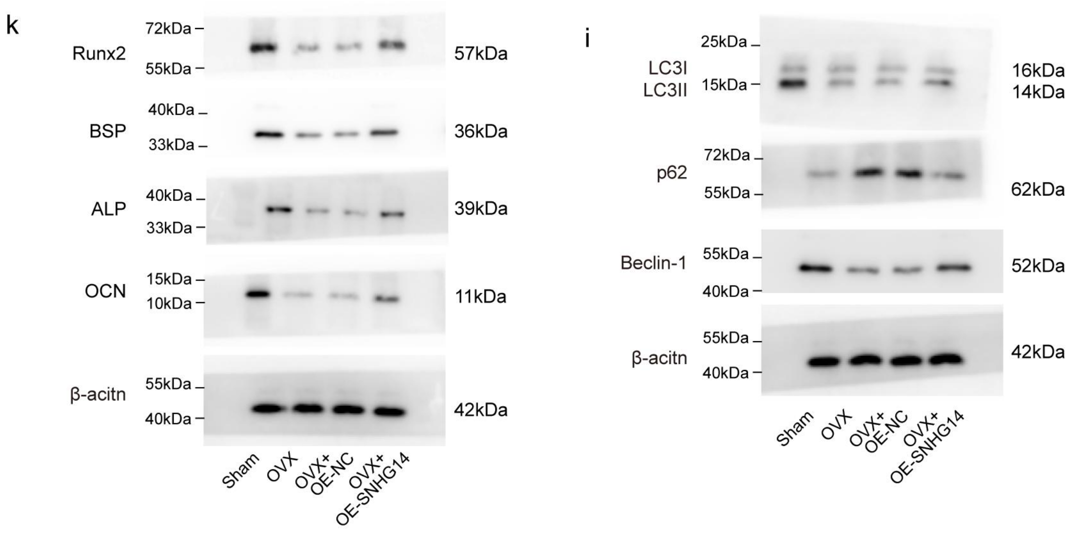

Supplementary Figure 6. Uncropped and unedited western blot images for Fig. 6.

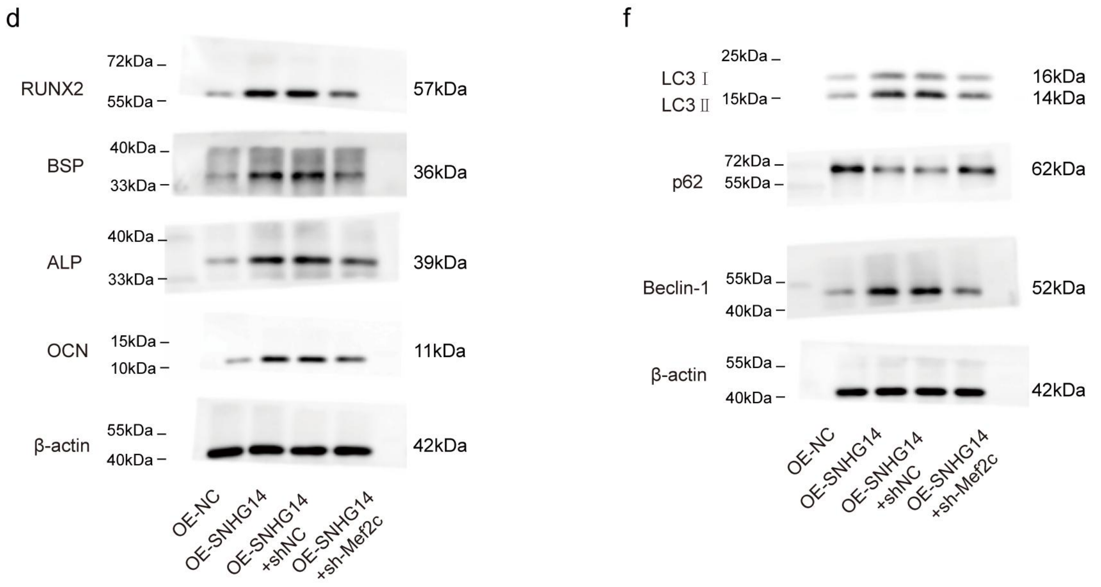

Supplementary Figure 7. Uncropped and unedited western blot images for supplementary Fig. 1.
